# Tremor-Suppression Orthoses for the Upper Limb: Current Developments and Future Challenges

**DOI:** 10.3389/fnhum.2021.622535

**Published:** 2021-04-30

**Authors:** Hoai Son Nguyen, Trieu Phat Luu

**Affiliations:** ^1^Group of Advanced Computations in Engineering Science, HCMC University of Technology and Education, Ho Chi Minh City, Vietnam; ^2^Noninvasive Brain-Machine Interface System Laboratory, Department of Electrical and Computer Engineering, University of Houston, Houston, TX, United States

**Keywords:** tremor treatments, upper-limb orthoses, wearable tremor-suppression orthoses, tremor extraction, medical device

## Abstract

**Introduction:** Pathological tremor is the most common motor disorder in adults and characterized by involuntary, rhythmic muscular contraction leading to shaking movements in one or more parts of the body. Functional Electrical Stimulation (FES) and biomechanical loading using wearable orthoses have emerged as effective and non-invasive methods for tremor suppression. A variety of upper-limb orthoses for tremor suppression have been introduced; however, a systematic review of the mechanical design, algorithms for tremor extraction, and the experimental design is still missing.

**Methods:** To address this gap, we applied a standard systematic review methodology to conduct a literature search in the PubMed and PMC databases. Inclusion criteria and full-text access eligibility were used to filter the studies from the search results. Subsequently, we extracted relevant information, such as suppression mechanism, system weights, degrees of freedom (DOF), algorithms for tremor estimation, experimental settings, and the efficacy.

**Results:** The results show that the majority of tremor-suppression orthoses are active with 47% prevalence. Active orthoses are also the heaviest with an average weight of 561 ± 467 g, followed by semi-active 486 ± 395 g, and passive orthoses 191 ± 137 g. Most of the orthoses only support one DOF (54.5%). Two-DOF and three-DOF orthoses account for 33 and 18%, respectively. The average efficacy of tremor suppression using wearable orthoses is 83 ± 13%. Active orthoses are the most efficient with an average efficacy of 83 ± 8%, following by the semi-active 77 ± 19%, and passive orthoses 75 ± 12%. Among different experimental setups, bench testing shows the highest efficacy at 95 ± 5%, this value dropped to 86 ± 8% when evaluating with tremor-affected subjects. The majority of the orthoses (92%) measured voluntary and/or tremorous motions using biomechanical sensors (e.g., IMU, force sensor). Only one system was found to utilize EMG for tremor extraction.

**Conclusions:** Our review showed an improvement in efficacy of using robotic orthoses in tremor suppression. However, significant challenges for the translations of these systems into clinical or home use remain unsolved. Future challenges include improving the wearability of the orthoses (e.g., lightweight, aesthetic, and soft structure), and user control interfaces (i.e., neural machine interface). We also suggest addressing non-technical challenges (e.g., regulatory compliance, insurance reimbursement) to make the technology more accessible.

## 1. Introduction

Neurological disorders are now globally the leading source of disability. Among neurological disorders examined in the Global Burden of Disease, Injuries, and Risk Factors Study (GBD) 2015, Parkinson's disease was the fastest growing in prevalence, disability, and deaths. In 2016, 6.1 million individuals were diagnosed with Parkinson's disease worldwide (Dorsey et al., 2018). Parkinson's disease is a chronic, slowly progressing degenerative disorder of the central nervous system and is characterized by the presence of resting tremor, rigidity, akinesia, and postural instability (Rocon and Pons, 2011). Pathological tremor is characterized by involuntary, rhythmic muscular contraction leading to shaking movements in one or more parts of the body (Anouti and Koller, 1995). Tremor is the most common motor disorder in adults and may be the consequence of neurological disease or drug use (Jankovic and Stanley, 1980; Deuschl et al., 1998; Bhidayasiri, 2005; Elble et al., 2013). Essential tremor (ET) and Parkinson's Disease (PD) are the two most prevalent conditions causing tremors in the upper limb and mostly affect the hands (Raethjen et al., 2000; Elble and Deuschl, 2011). Though tremor is not life-threatening, it can be frustrating and even disabling, causing serious difficulties in performing daily activities (National Institute of Neurological Disorders and Stroke, 2020).

A pathological tremor in the upper limb can be categorized into two main types: resting (or static) tremor and action tremor. Resting tremor occurs when a body part is relaxed and completely supported against gravity. It is typically amplified by mental stress or movement of another part of the body, and reduced by voluntary movement of the affected body part (Bhidayasiri, 2005). Most tremors are action tremors, which occur with voluntary contraction of a muscle. Action tremors can be further subdivided into postural, isometric, and kinetic tremors (Crawford and Zimmerman, 2011). Postural tremor is present while maintaining a position against gravity. Isometric tremor occurs with muscle contraction against a rigid stationary object. A kinetic tremor is associated with any voluntary movement and includes intention tremor, which is produced with target-directed movement (Deuschl et al., 1998). While resting tremors are a cardinal feature in patients with PD, action tremor in the upper limb(s) is the main symptom in ET, especially the kinetic tremor (Cohen et al., 2003; Jankovic, 2008; Thenganatt and Louis, 2012).

Pharmaceutical medication is one of the most commonly used treatments against PD and ET tremors. Unfortunately, it is not effective in treating tremor and carries significant negative side effects. For example, tremor is not controlled effectively or adequately in about 25% of patients (Gallego et al., 2010). The side effects from medication include allergic reactions, nausea, heart problems, reduction of white blood cells, etc. (Matsumoto et al., 2013). As a result, more than one-half of people discontinue medical treatment due to side-effects or lack of efficacy (Diaz and Louis, 2010; O'Connor and Kini, 2011). The use of electrical stimulation to a specific part of the brain (Deep Brain Stimulation, DBS) has emerged as one of the most effective treatments for most tremors. For example, while the tremor reduction efficacy of the medication treatment range from 23 to 59% for PD (Koller, 1986), the efficacy is 90% for DBS method (Elble and Deuschl, 2011). However, DBS is an invasive treatment and it carries the risk of surgical complications (the implantation of electrodes into the brain) (Katayama et al., 2005; Hariz et al., 2008).

Considering the drawbacks of the traditional treatments for tremor (e.g., the lack of efficacy, side-effects from medication, potential risks from brain surgery), there is a significant need for effective, non-invasive tremor treatments. Recent approaches include the EU TREMOR project which aimed to apply selective biomechanical loads through multichannel array FES to compensate tremors without impeding the voluntary movements from the user (Pons, 2011). Prior studies also reported the promise of using a closed-loop FES to activate tremorogenic muscles out-of-phase to counteract the tremor (Elek and Prochazka, 1989; Javidan et al., 1990). However, the major challenges, such as the misalignments of the FES electrodes due to movements and muscle fatigue must be resolved for the long-term use of FES devices (Tepavac and Schwirtlich, 1997). The Movement Disorder Society Evidence-Based Medicine Panel also recommended exercise and physical therapy as an effective alternative to the traditional tremor treatments (Fox et al., 2011). The short-term and, to a lesser extent, long-term benefits of physical therapy interventions in treating tremor in patients with PD have been demonstrated in the last decades (Allen et al., 2011; Shen et al., 2016). Additionally, recent studies have demonstrated the effectiveness of the biomechanical loading method using wearable orthoses for tremor suppression (Adelstein and Rosen, 1981; Rocon et al., 2005; Kazi et al., 2010; Taheri et al., 2013; Zhou et al., 2017). Such devices usually comprise advanced signal processing algorithms to estimate voluntary and tremorous movements from biomechanical sensors [e.g., joint angles, acceleration, interaction force, surface Electromyography (sEMG) and a suppression mechanism that generates mechanical loads to attenuate tremorous movements].

In this review paper, we will summarize the state of the art of upper-limb wearable orthoses for tremor suppression. The summary includes both mechanical development and control strategies. This review also aims to identify the limitations in current orthoses in tremor treatment and to discuss potential research directions for improvement in future design and development.

## 2. Methods

### 2.1. Search Methods and Keywords

**Figure 1:**
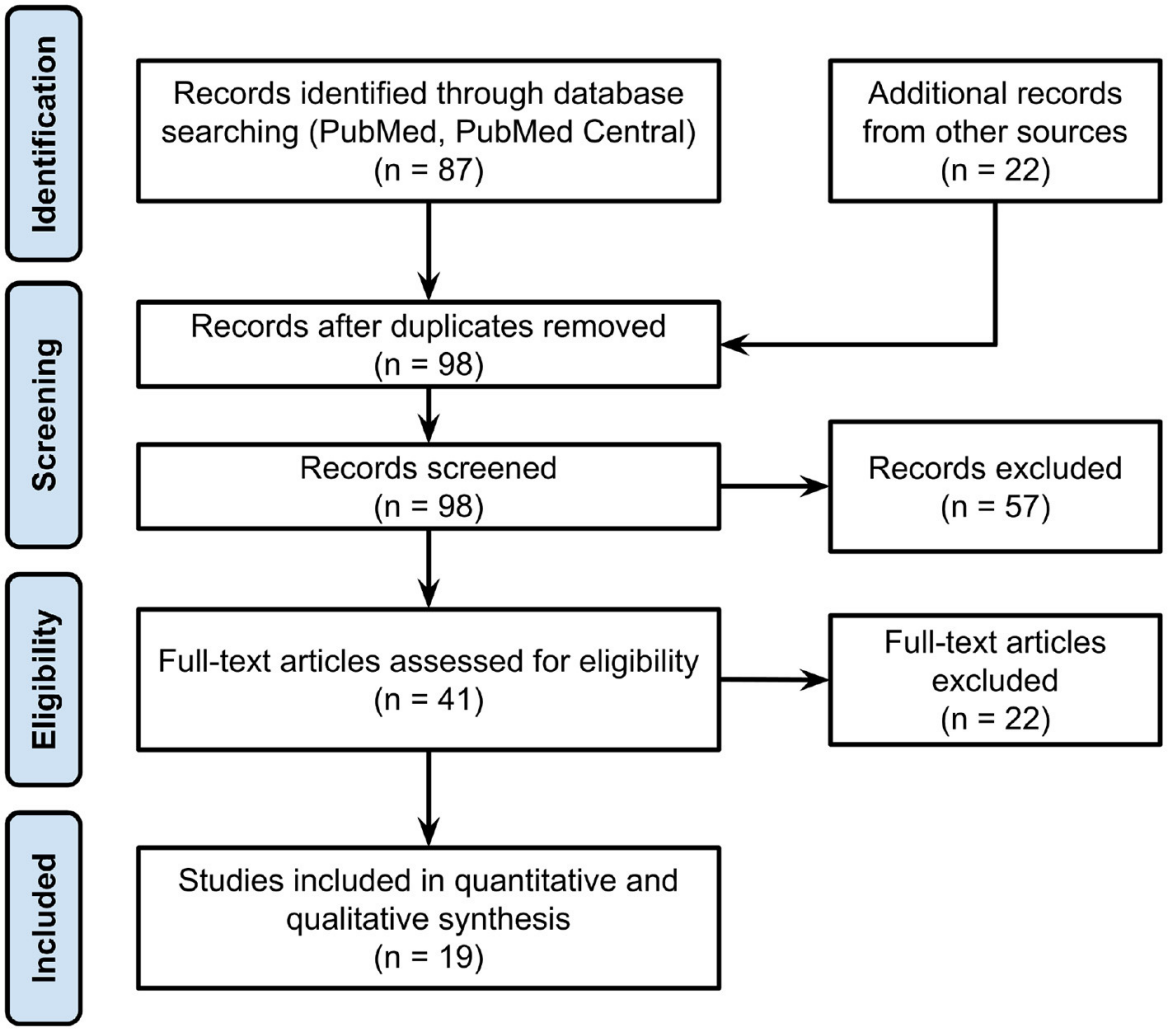
PRISMA flow chart of the method used for articles' selection.

We followed the Preferred Reporting Items for Systematic Reviews and Meta-analysis (PRISMA) to search and screen for relevant studies in this review article. Details of the PRISMA flow chart are illustrated in Figure 1. To approach the *Identification* stage systematically, we wrote a Python script and utilized NCBI Entrez API to conduct the literature search. The number of studies were limited to 200 items and only accepted the studies within the last 50 years. The search keywords were applied to query data from the PMC and PubMed databases: *(“tremor management” OR “pathological tremor” OR “tremor assessment” OR “essential tremor” OR “pathological tremor” OR “tremor suppression” OR “tremor treatment” OR “tremor canceling”) AND (exoskeleton OR orthosis OR “assistive robot” OR “assistive device”) NOT (“electrical stimulation”[title] OR “surgical”[title] OR “multiple sclerosis”[title])*. We performed the query and identified 87 articles on May 15, 2020. Additional records were obtained from other database and search engine (e.g., Google Scholar, IEEE) using the same keywords. Duplicates and studies that did not meet the inclusion criteria (see below) were excluded. The two reviewers obtained and screened full texts of the remaining relevant studies and compared the results. In the case of disagreement, the last reviewer (TPL) was decisive.

### 2.2. Criteria for Selection of Studies

The inclusion criteria for a study to be included in the review are as follows:

- Upper-limb exoskeleton or orthoses
- Passive or active orthosis for tremor suppression
- Wearable devices for tremor suppression at musculoskeletal level
- Wearable tremor-suppression orthoses
- Tremor treatment was controlled exclusively by the uses of upper-limb orthoses without pharmacological treatments.

Exclusion criteria:

- Fixed/grounded upper-limb exoskeleton
- Lower-limb exoskeleton
- Functional Electrical Stimulation (FES) for tremor suppression
- Tremor treatments not using biomechanical loading [e.g., Deep Brain Stimulation (DBS), drugs, surgery].

### 2.3. Data Extraction

The remaining studies that meet all of the inclusion criteria in section 2.2 and are eligible for full-text access will be further analyzed. We extracted the following information from each study:

- Suppression mechanisms
  The types of mechanism (i.e., passive, semi-active, active) that were implemented to suppress tremorous movements (details in section 3.1)
- Degrees of Freedom (DOFs) and weights of the orthoses
  DOFs represent the number of upper limb joints in which the involuntary movements are suppressed by an orthosis. The weight of an orthosis is a crucial factor that impacts the usability and functionality of the orthosis, as well as the acceptability from users. In this review paper, the weights of external systems that are not carried by the users, such as battery power supply were not included.
- Efficacy of tremor suppression
  The efficacy was computed by comparing tremor measurements with and without the suppression from an orthosis. The comparison metrics could be in the frequency domain or in the temporal domain. The efficacy values were converted to a percentage (%) for comparison across different studies. Three different types of experimental setups include (1) bench testing without human subjects, (2) healthy individuals, and (3) subjects with tremor-affected.
- Tremor measurements
  The type of sensors that were used to measure physiological and biomechanical data from a user. For example, Inertial Measurement Unit (IMU), force sensors, sEMG, etc.
- Algorithms for the estimation of voluntary and tremorous movements
  Signal processing methods and algorithms for extracting tremorous movements from voluntary movements.

### 2.4. Degrees of Freedom (DOFs) of Human Upper Limb

**Figure 2:**
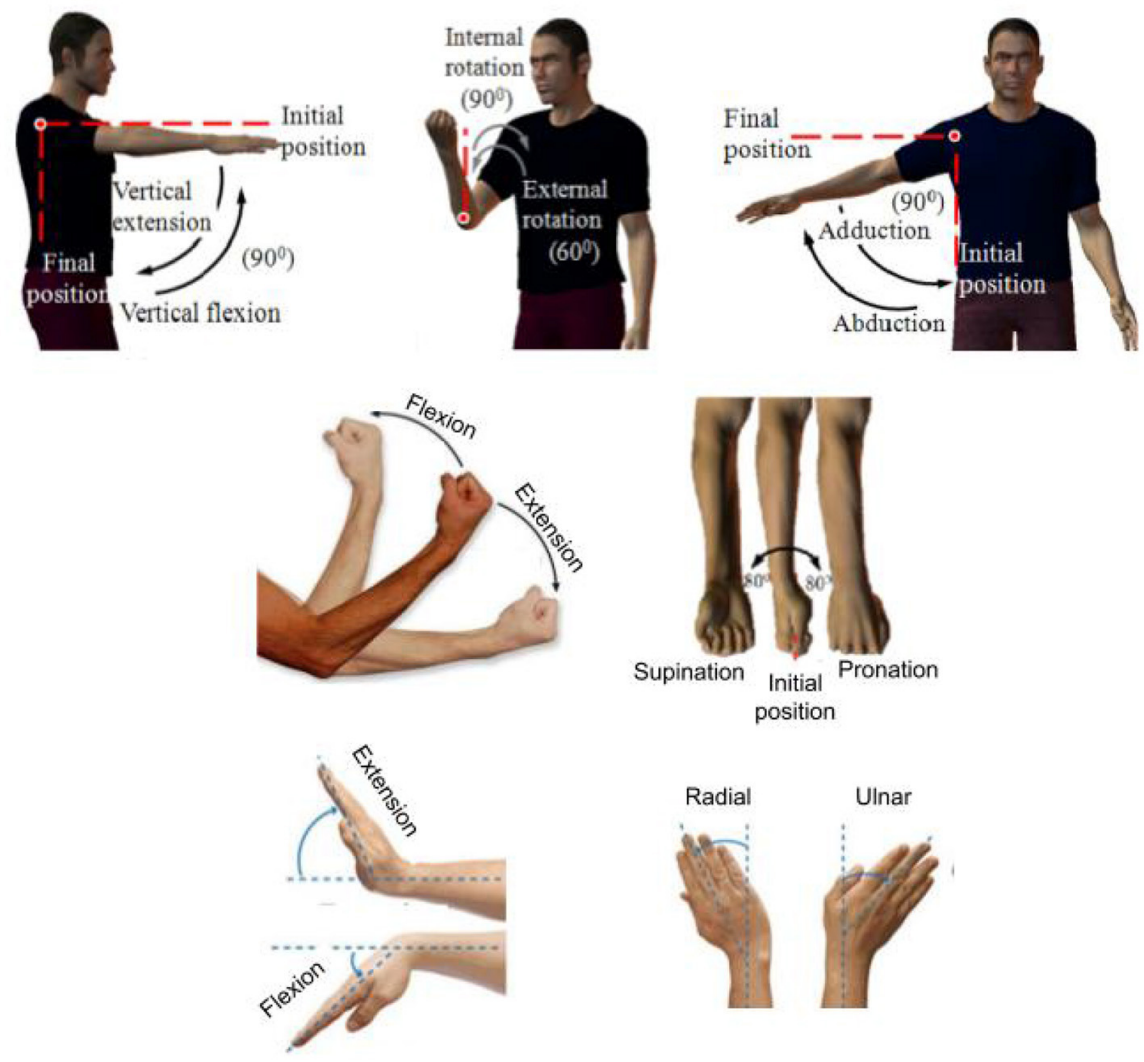
Seven DOFs of human upper limb (excluding the hand). Adopted and modified from Gopura and Kiguchi (2009).

The kinematic model of an upper-limb (without considering the DOFs in the hand) has seven DOFs: shoulder flexion/extension, shoulder adduction/abduction, shoulder internal/external rotation, Elbow Flexion/Extension (EFE), Forearm Pronation/Supination (FPS), Wrist Flexion/Extension (WFE), and Wrist Radial/Ulnar Deviation (WRU). The shoulder DOFs were omitted in this review paper because none of the identified studies considered tremor suppression for this joint. The illustration of motions of human upper limb (7 DOFs) is shown in Figure 2.

## 3. Results

We applied the PRISMA flow chart to screen the records downloaded from PubMed, PMC, and other resources (e.g., IEEE, Google Scholar) and obtained 19 articles that met the inclusion and full-text criteria. The results are shown in Table 1 and the tremor-suppression orthoses are grouped based on the suppression mechanism. In particular, the light coral, light blue, and light gray background colors represent the active, semi-active, and passive orthoses, respectively.

**Table 1:** Upper-limb orthoses for tremor suppression.

**References**	**Device**					**Testing**	
	**Name/Group**	**Actuator**	**Weight (g)**	**DoF**	**Efficacy (Metrics)**	**Subjects**	**Bench test^a^**
Rocon and Pons (2011)	WOTAS	DC motor	850	3 (EFE, FAA, WFE)	40% (PSD)	10 (ET, PD, MS)	0
Ando et al. (2012)	EMG-controlled Exo	DC motor	330	1 (EFE)	−	1 ET; 4 HT	0
Matsumoto et al. (2013)	Voluntary-driven elbow orthosis	DC motor	410	1 (EFE)	50–80% (AA)	1 ET 1 HT (FES)^b^	0
Huen et al. (2016)	ADL Exo	Servo m͡otor	350	2 (FPS, WFE)	77% (AA)	6 HT (VB, 3 Hz)^c^	0
Zhou et al. (2017)	MMS	DC motor	129	1 (WFE)	−	0	7 PD
Zhou et al. (2018)	WTSG	DC motor	320–340	3 (WFE, Index, Thumb)	85 ± 8% (JA) 88 ± 14% (PSD1) 92 ± 7% (PSD2)	0	7 PD
Herrnstadt et al. (2019)	TSO	DC motor	1,700	1 (EFE)	94.4% (PSD)	9 (PD, ET)	0
Zamanian and Richer (2019)	PMLM	Linear motor	−	2 (WFE, WUD)	97% (PSD)	0	10 (PD, ET)
Wang et al. (2019)	TAWE	Servo motor	485	2 (WFE, WUD)	−	−	−
Loureiro et al. (2005)	DVB	MR damper	≈200	2 (WFX, WAA)	43 dB (PSD)	1 ET	0
Herrnstadt and Menon (2012)	EFB	Electro-magnetic brake	942	1 (EFE)	88% (PSD)	3 HT (SI, 3–4 Hz)^d^	0
Taheri et al. (2014)	Southern Methodist University	Pneumatic cylinder	−	2 (WFE, WAA)	98.8% (PSD)	0	10 (PD, ET)
Case et al. (2015)	MR damper	MR damper	−	4 (EFX, FPS, WFE, WAA)	32 ± 2.5 dB (PSD1) 13.7 ± 5.7 dB (PSD2)	0	10 (PD, ET)
Yi et al. (2019)	WTSE	MR damper	262	1 (WFE)	60.39% (AA)	5 HT (SI, 2–4 Hz)	0
Zahedi et al. (2020)	SETS	MR damper	255	2 (WFE, WUD)	61.39%	5 HT (SI, 2–8 Hz)	−
Kotovsky and Rosen (1998)	Viscous beam	Constrained-layer-damping	260	1 (WFE)	−	−	−
Takanokura et al. (2011)	Air dashpots	Air dashpots	−	3 (EFE, WFE, WUD)	20–62%	1 HT (FES)	−
Buki et al. (2018)	Vib-bracelet	Tuned mass damper	280	1 (FAA)	85%	12 PD	−
Fromme et al. (2020)	TAPO	Air-filled	33	2 (WFE, WUD)	78%	1 PD	−

*EFE, elbow flexion/extension; FPS, forearm pronation/supination; WFE, wrist flexion/extension; WRU, wrist radial/ulnar deviation; JA, joint angle amplitude; AA, acceleration amplitude; PSD1, PSD2, spectral power at the 1st and 2nd harmonic*.

a *Bench Testing without human subject. Tremorous input motion was used from recorded datasets*.

b *Healthy subject. FES was applied to simulate tremorous movements*.

c *Healthy subject. Mechanical vibration was applied*.

d *Healthy subject. Self-initiated oscillation movements*.

*Colors represent different suppression mechanisms (light red: Active; light blue: Semi-active; light gray: Passive)*.

### 3.1. Suppression Mechanisms

Biomechanical loading using wearable orthoses for tremor suppression can be categorized into three types based on the suppression mechanism: passive, semi-active, and active systems. A passive system adopts shock absorbers attached to the tremor-affected upper limb for tremor suppression. A semi-active system estimates the level of tremor based on measurements from sensors and uses the information to suppress movements by updating the impedance of the system. The semi-active mechanism also suppresses tremorous motions by absorbing energy. While the passive and semi-active mechanisms attenuate tremor motion by absorbing energy, the active mechanism can actively provide motion in the opposite direction to restrict the tremor motions.

**Figure 3:**
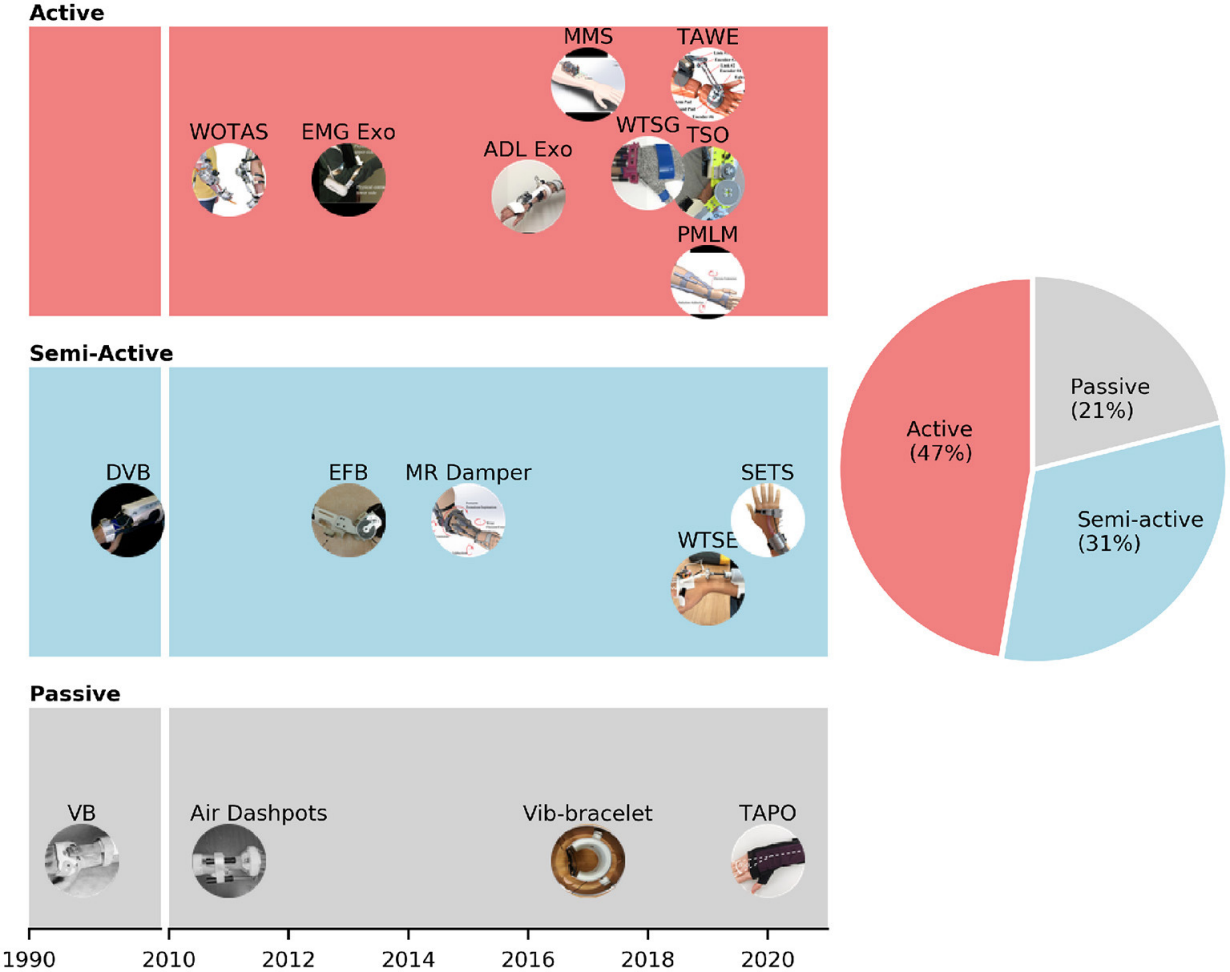
Different suppression mechanisms (passive, semi-active, and passive) of wearable orthoses for tremor suppression.

Figure 3 shows the developmental progress of different types of suppression mechanisms. The majority of wearable orthoses for tremor suppression are active with 47% prevalence. In the past 10 years, active systems for tremor suppression have gained increasing levels of attention from research groups around the world. Electric DC and servo motors are primarily used in active devices as actuators which drive the upper-limb joints via a gear box or cable transmission system. Among the active devices, WOTAS (Wearable Orthosis for Tremor Assessment and Suppression) was the first to be developed and the most well-known orthosis for upper-limb tremor suppression (Rocon et al., 2007). The WOTAS development followed the framework of the Dynamically Responsive Intervention for Tremor Suppression) (DRIFTS) project (Manto et al., 2004). WOTAS was developed with three main objectives: monitoring, diagnosis, and validation of non-grounded tremor reduction strategies (Rocon et al., 2005). Semi-active mechanism accounts for 31% of the devices founded in this review paper (Table 1). Semi-active mechanisms are primarily relied on MagnetoRheological (MR) fluid to produce the damping force (Loureiro et al., 2005; Case et al., 2015; Yi et al., 2019; Zahedi et al., 2020). When exposed to a magnetic field, the viscosity of MR fluid can be modulated by the magnetic strength field. MR fluid has recently received increased levels of interest in the field of wearable exoskeletons because of its high strength-to-weight ratio property (Perry et al., 2007). Semi-active wearable devices using MR fluid dampers can be considered as promising solutions for tremor suppression (Tsang et al., 2006). Passive mechanisms cover the last 21% of the wearable orthoses. Viscous beam, which was developed by Kotovsky et al. in 1998, was the first passive device designed for tremor suppression purposes (Kotovsky and Rosen, 1998). Task-Adjustable Passive Orthosis (TAPO) is the most recent device in this category (Fromme et al., 2020). Fromme et al. designed this orthosis based on an air-filled structure and achieved a very compact and lightweight device (33 g).

### 3.2. Degrees of Freedom and Weights of the Wearable Orthoses

**Figure 4:**
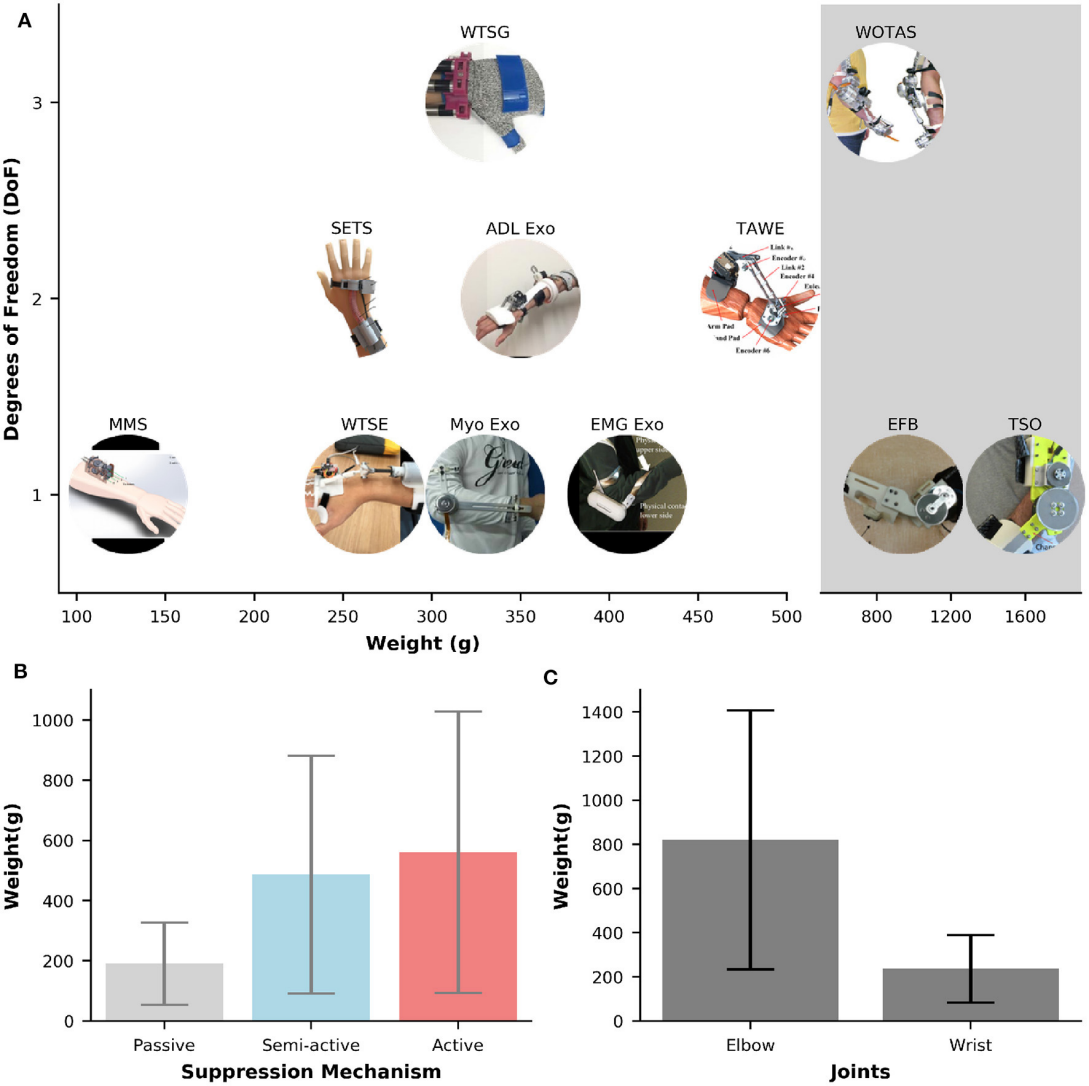
**(A)** Overview of weights and numbers of DOF for semi-active and active orthoses. The shaded gray area represents the systems that weight larger than >850 g. **(B,C)** Bar plots of weights against different suppression mechanisms and joints, respectively. Error bars represents one standard deviation.

Figure 4A illustrates the weights and DOFs of semi-active and active devices for tremor suppression. The majority of the orthoses (54.5%) only support one DOF. Two-DOF and three-DOF orthoses account for 33 and 18%, respectively. Among the one-DOF orthoses, four out of six were designed to support the tremor control at the elbow joint (EFE) (Ando et al., 2010; Herrnstadt and Menon, 2012; Matsumoto et al., 2013; Herrnstadt et al., 2019), and the others were developed to suppress the WFE motions (Zhou et al., 2017; Yi et al., 2019). Interestingly, all of the two-DoF orthoses are semi-active and support tremor control at the wrist joint (WFE and WUD) (Huen et al., 2016; Wang et al., 2019; Zahedi et al., 2020). Three-DOF orthoses shown in Figure 4A include WOTAS and Wearable Tremor Suppression Glove (WTSG). While WOTAS provides tremor suppression at the elbow (EFE), forearm (FPS), and wrist (WFE) (Rocon and Pons, 2011), WTSG was developed for tremor suppression at the wrist (WFE), finger, and thumb joints (Zhou et al., 2018).

The most common DOFs for suppression are the WFE and EFE, which can be found in ~64%, and 45% of the orthoses, respectively. Tremor suppression at the FPS, finger and thumb joints are only available in 9% of the orthoses. No system is found to only suppress the tremor for WUD. While the mechanisms for tremor suppression at WFE can be semi-active or active, all of the mechanisms for EFE are active except for the EFB orthosis which relied on the electromagnetic brake for motion damping. Active orthoses for EFE tremor control are typically powered by strong DC motors.

In general, the weight of an orthosis increases proportionally to the number of DoFs to be suppressed. Table 1 shows a large variation in the orthoses weights for tremor suppression, ranging from 33 g [TAPO, passive device, air-filled structure (Fromme et al., 2020)] to 1,600 g [Tremor Suppression Orthosis (TSO), active, DC motor (Herrnstadt et al., 2019)]. The average weight of an orthosis is 456 (±409 g). The Multichannel Mechatronic Splitter (MMS) developed by Zhou et al. (2017) is the most compact and lightweight active orthosis found in this study. This device weighs about 129g and is powered by a small 2W DC motor and uses cables for transmission. The shaded area in Figure 4A illustrates the orthoses that, due to their high weight (>800 g), make the completion of everyday activities a challenge. Specifically, the weights in the shaded area are 850 g [WOTAS, active, DC motor (Rocon and Pons, 2011)], 942 g [EFB, semi-active, electromagnetic brake (Herrnstadt and Menon, 2012)], and 1,600 g [TSO, active, DC motor (Herrnstadt et al., 2019)].

The weight of an orthosis does not only depend on the number of DOFs but also its type of tremor suppression mechanisms (i.e., passive, semi-active, active). Figure 4B shows the weight values against different types of suppression mechanisms. The passive orthoses have the smallest average weight of 191 (±137 g), followed by the semi-active orthoses with the average weight of 486 (±395 g). Active orthoses are the heaviest with an average weight of 561 (±467 g). The weights of orthoses also vary across different types of DoF to be suppressed. Figure 4C shows the average weights of orthoses that were developed specifically for elbow or wrist suppression. The average weight of an orthosis for tremor suppression at the wrist joint is 237 (±152 g), ranging from 33 to 485 g. This value is approximately two times larger in the orthosis that only supports the elbow joint. Specifically, the average weight of the orthoses that support the elbow joint is 821 (±586 g), ranging from 330 to 1,600 g.

### 3.3. Efficacy of Tremor Suppression Using Wearable Orthoses

The experimental settings to evaluate the tremor suppression performance of an orthosis can be categorized into three groups: bench testing (without human subject), healthy individuals, and individuals with tremor disorders. In the bench testing setup, a separate system is built and connected to a wearable orthosis. The tremorous motions are simulated by playing back different types of tremor (i.e., PD, ET) from available clinical datasets. For example, the dataset from Timmer et al. (2000) including tremorous motion data from ten patients (5 PDs, 5 ETs) has been used in several studies for bench testing. A tremorous motion can also be simulated by defining its amplitude and dominant frequencies (e.g., 3–12 Hz) and inducing it with a signal generator. In the experimental setup that involves healthy individuals with no history of neurological disease and pathological tremor, the subjects were instructed to self-initiate tremorous motions by swinging their wrist at a certain frequency (Yi et al., 2019; Zahedi et al., 2020). Involuntary tremor stimulation on healthy individuals can also be induced by using FES. The FES control parameters can be set to stimulate tremors at different frequencies (e.g., 3 and 5 Hz for PD and ET tremors, respectively) (Takanokura et al., 2011). Finally, the experimental setup with tremor-affected (impaired) individuals recruited subjects who were diagnosed with pathological tremors (e.g., PD, ET).

**Figure 5:**
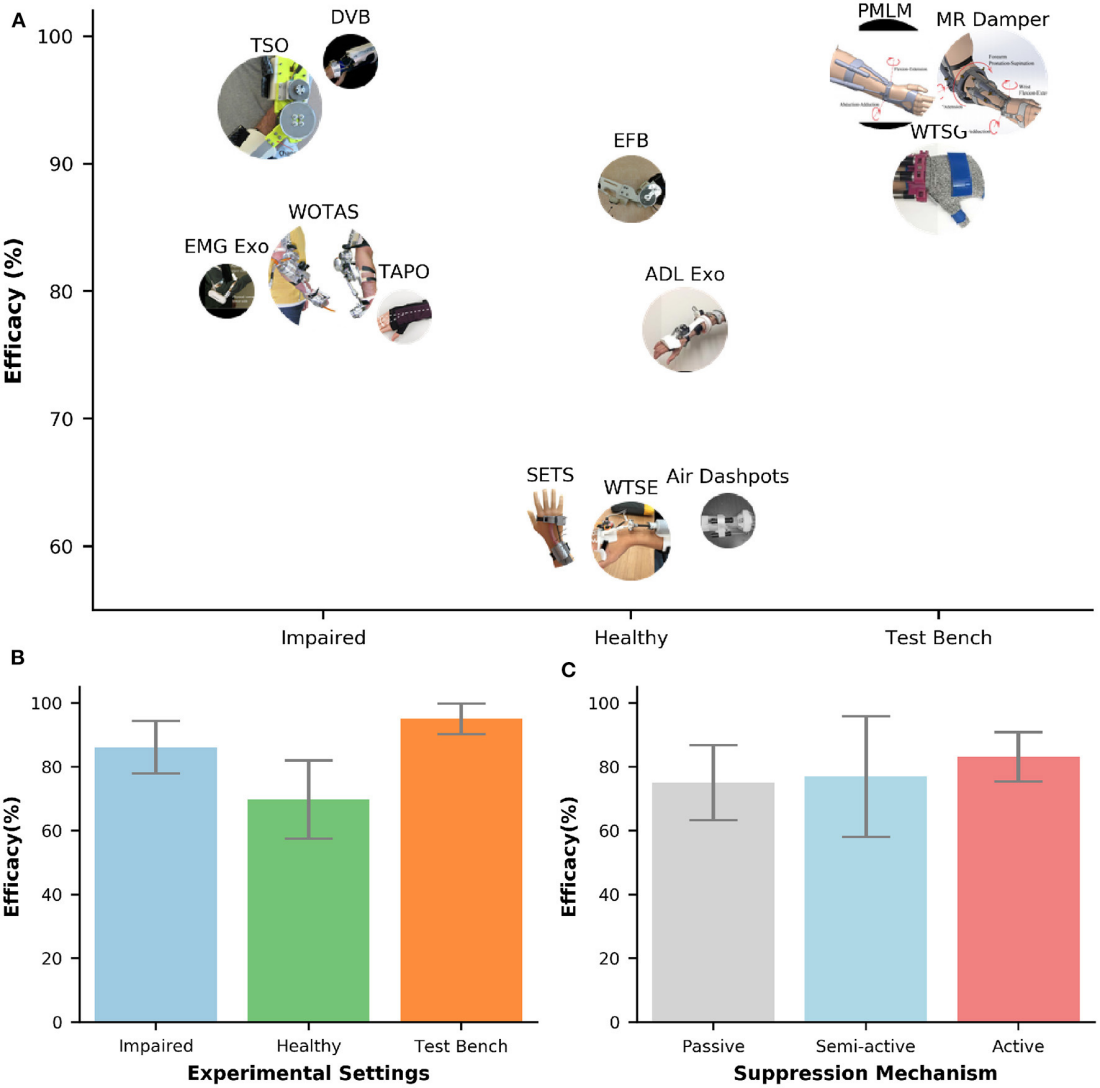
**(A)** Efficacy of tremor-suppression orthoses under different types of experimental setups (bench test, healthy, and impaired). The circle's size represents the number of participants. **(B,C)** Bar plots of efficacy against experimental settings and suppression mechanisms, respectively. The error bars represent one standard deviation.

Figure 5A shows the efficacy of different orthoses. The efficacy was computed by comparing biomechanical data (e.g., joint angles, velocities, acceleration, interaction forces, sEMG) under two conditions: with and without tremor suppression from the orthoses. The comparison could be performed by using metrics in the frequency domain [e.g., Power Spectral Density (PSD), dominant harmonic frequencies] or in the temporal domain [e.g., amplitudes, Root Mean Square (RMS)]. The efficacy values in Figure 5 were converted to percentages (%) for comparison across different studies. The majority of the orthoses (77%) in Figure 5 were evaluated with human subjects. Only two semi-active (Case et al., 2015; Zamanian and Richer, 2019) and one active orthosis (Zhou et al., 2018) were assessed with bench testing. All passive devices found in this review performed the tremor suppression assessment on human subjects (Takanokura et al., 2011; Fromme et al., 2020). The average efficacy of tremor suppression using wearable orthoses is 83 (±13%). The lowest efficacy is 60% (Wrist Tremor Suppression Exoskeleton (WTSE), tested with five healthy individuals) (Yi et al., 2019), and the highest efficacy is 98% (DVB, tested with 1 ET) (Loureiro et al., 2005).

Figure 5B shows the bar plots of efficacy under different experimental settings. Among those setups, bench testing shows the highest efficacy of tremor suppression and the smallest deviation of 95 (±5%). On the other hand, the lowest efficacy value and the highest deviation is found on testing with healthy individuals 70 (±12%). The average efficacy for the orthoses evaluated with tremor-affected subjects is 86 (±8%). The efficacy of tremor suppression also varied across different types of tremor suppression mechanisms (Figure 5C). The active orthoses have the largest average efficacy and the smallest deviation of 83 (±8%), followed by the semi-active orthoses with the average efficacy of 77 (±19%). Passive orthoses have the lowest average efficacy of 75 (±12%).

### 3.4. Tremor Measurements and Algorithms for the Estimation of Tremorous Movements

The upper-limb activities of individuals with pathological tremors are comprised of both voluntary and tremorous movements. To optimize the efficacy of tremor suppression in a semi-active or active orthosis, an intuitive controller is desired to suppress only tremorous movements without affecting the voluntary movements (Taheri et al., 2014). The challenges for developing such controllers include but are not limited to the estimation of pathological tremors with high accuracy and robustness from wearable sensors. Additionally, the controller is also expected to maintain its performance in real-time with minimum time delays.

#### 3.4.1. Characteristics of Parkinsonian and Essential Tremors

Optimizing tremor control using an assistive orthosis requires understanding the characteristics of pathological tremors. The voluntary and tremorous movements are usually characterized by their frequency contents. The prominent frequency of tremors could be visible and approximated by the naked eye. However, more accurate quantification requires measured data (e.g., sEMG, force sensor, IMU) and proper signal processing methods. Prior studies have shown that the voluntary motion for most Activities of Daily Living (ADL) have a frequency spectra below 2 Hz (Mann et al., 1989,?; Rocon et al., 2007; Gallego et al., 2009). Meanwhile, the pathological tremors are reported to have their spectral energy concentrated in the 3–12 Hz range (Stiles and Randall, 1967; Elble and Randall, 1978; Anouti and Koller, 1995; Deuschl et al., 1998; Charles et al., 1999; Ang et al., 2001; Loureiro et al., 2005; Engin, 2007; Heldman et al., 2011). Some recent studies have reported varied results in which the Parkinsonian tremor (PD) and the essential tremor (ET) contain the frequencies from 3.5 to 17.3 Hz (Taheri et al., 2013; Zhou et al., 2016). Despite the consensus about the frequency range of pathological tremors, it is challenging to distinguish between the PD and the ET tremors (Puschmann and Wszolek, 2011; Thenganatt and Louis, 2012). A study from Zhang et al. with 45 patients (25 PD and 20 ET) showed considerable overlap between the tremor frequency in the PD group (4–6 Hz) and in the ET group (5–8 Hz) (Zhang et al., 2017). Burne et al. conducted a quantitative analysis of acceleration and sEMG data from 42 patients with tremor (22 PD and 20 ET) and reported similar results. For example, this study showed that more than 95% of PD group exhibited frequencies within a narrow 4–6 Hz band, and 95% of ET patients occurred in the 5–8 Hz range.

Prior studies also reported similar frequency bands associated with PD tremors; for example, (4–6 Hz) (Cooper and Rodnitzky, 2000), (3.5–6 Hz) (Smaga, 2003), and (3–5 Hz) (Puschmann and Wszolek, 2011). The frequency band of ET tremor however, varies across different studies. While Javidan et al. (1992) showed a relatively narrow frequency band of (5–8 Hz) for ET tremor, Cooper and Rodnitzky (2000) and Elble (2000) reported ET tremor frequency band at (4–12 Hz) and (3–11 Hz), respectively. ET tremor was also reported in the frequency band at (4–10 Hz) (Ando et al., 2010; Puschmann and Wszolek, 2011; Matsumoto et al., 2013; Hassan et al., 2016).

#### 3.4.2. Algorithms for the Real-Time Estimation of Voluntary and Tremorous Movements

**Figure 6:**
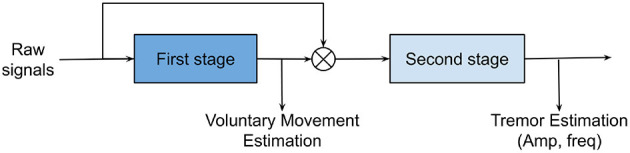
Two-stage tremor modeling. Voluntary motion in the low-frequency bands is estimated in stage 1. Subsequently, stage 2 will characterize tremorous motions (i.e., amplitude and frequencies) after subtracting the voluntary motions from the original motions.

A tremor modeling method should be able to extract voluntary movements and to quantify tremorous movements (i.e., in both amplitude and frequency contents) with minimum time delay. Tremor extraction algorithms may include one or multiple stages. Rocon and Pons (2011) developed a two-stage model for tremor extraction which is illustrated in Figure 6. In two-stage modeling, the first stage is responsible for estimating volitional movements from physiological input signals and the second stage characterizes the tremorous movements.

A summary of sensors and algorithms in tremor-suppression orthoses is shown in Table 2. The majority of the orthoses (92%) measured voluntary and/or tremorous motions using biomechanical sensors (e.g., IMU, force sensor). Only one system from Ando et al. (2010) utilized surface EMG for tremor extraction.

**Table 2:** Algorithms for voluntary and tremor estimation.

**References**	**Name/Group**	**Sensors**	**Voluntary estimation**	**Tremor estimation**	**Controller**
Zahedi et al. (2020)	SETS	IMU	–	BPF (2–14 Hz)	PD controller. Time delay < 0.1 s
Herrnstadt et al. (2019)	TSO	Torque	–	LPF + ABPF	uter loop: PID admittance. Inner loop: PI velocity control.Sampling rate at 100 Hz
Yi et al. (2019)	WTSE	IMU	–	BPF (2–14 Hz)	PD controller. Time delay < 0.1 s.
Zamanian and Richer (2019)	PMLM	IMU	–	HPF + BPF + AFE	Virtual model control
Zhou et al. (2018)	WTSG	IMU	–	WFLC	The control strategy is to follow the voluntary movements of the user.
Herrnstadt and Menon (2016)	TSO	Torque	Kalman Filter	–	Outer loop: PID force control. Inner loop: PI velocity control.
Huen et al. (2016)	ADL Exo	IMU	–	–	On-off control using threshold of motion frequency at 3 Hz
Case et al. (2015)	MR damper	IMU	–	HPF + BPF + AFE	PID to control damping force
Matsumoto et al. (2013)	–	IMU	–	BPF (4–6 Hz)	Manual control with toggle on-off switch
Herrnstadt and Menon (2012)	EFB	Gyros + potentionometer	–	HPF (2 Hz)	On-Off control of the electromagnatic brake when tremor was detected in PSD plot
Ando et al. (2012)	EMG Exo	EMG	STFT + TDNN	–	On-Off control to assist the voluntary movement
Rocon et al. (2007)	WOTAS	IMU + Torque	Benedict-Bordner filter	WFLC	• Impedance control • Notch filtering at tremor frequency
Loureiro et al. (2005)	DVB	IMU	Benedict-Bordner filter	WFLC	Control viscous damping based on tremor estimation

*LPF, low pass filter; HPF, high pass filter; BPF, band pass filter; ABPF, adaptive band pass filter; AFE, adaptive frequency estimator; WFLC, weighted-frequency Fourier linear combiner; STFT, short-time Fourier transformation; TDNN, time delay neural network*.

**Figure 7:**
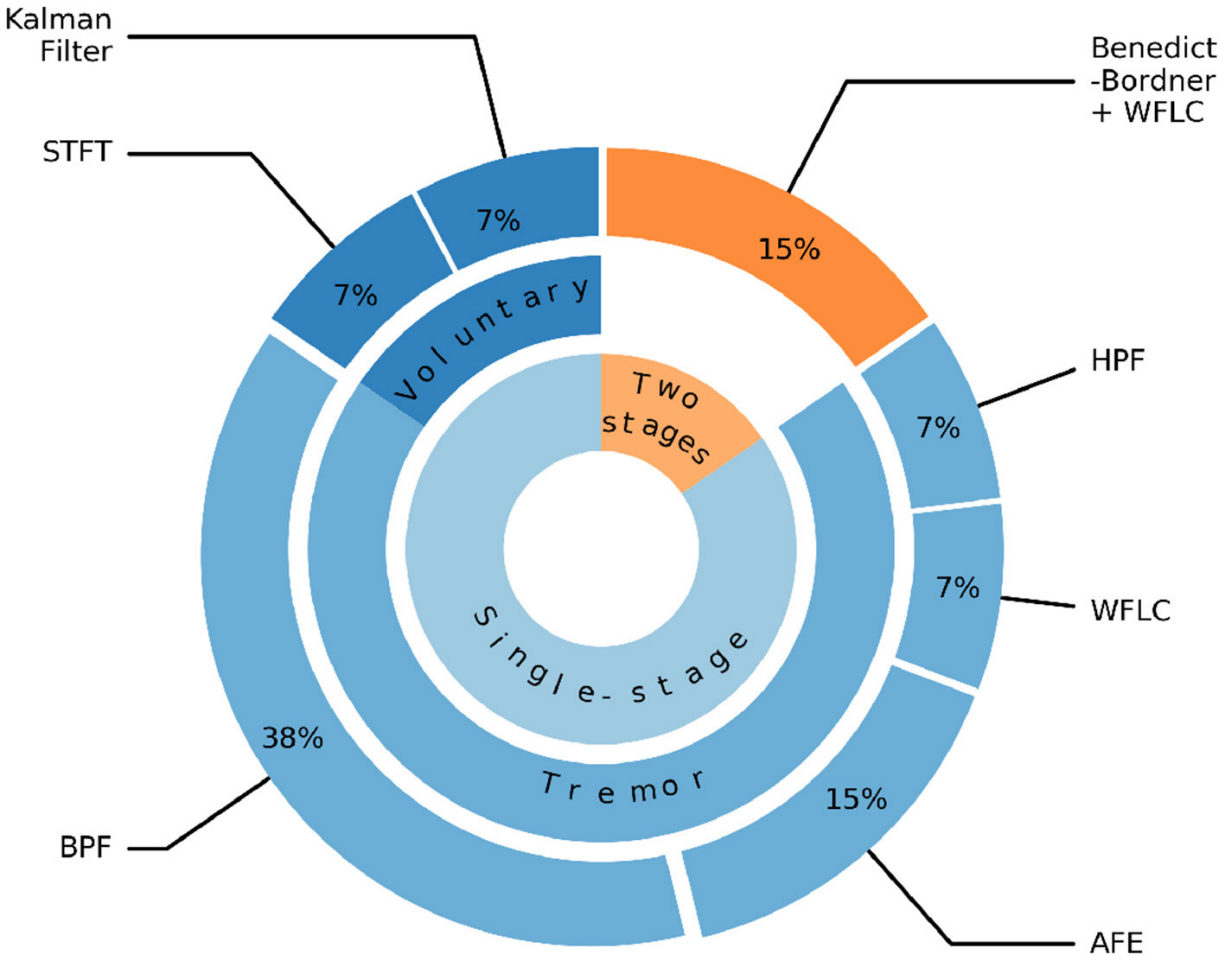
Algorithms for extracting voluntary and tremorous movements. STFT, Short-time Fourier Transform; LPF, Low-pass filter; BPF, Band-pass filter; HPF, High-pass filter; WFLC, Weighted Frequency Fourier Linear Combiner; AFE, Adaptive Frequency Estimator.

Figure 7 illustrates that the majority of tremor estimation algorithms consist of a single-stage 85%. In the single-stage method, the filtering algorithm could be used to extract voluntary movement [e.g., Kalman filter, Low-pass Filter (LPF), Short-time Fourier Transform (STFS)/Time Delay Neural Network (TDNN)], or tremorous movement [e.g., High-pass Filter (HPF), Band-pass Filter (BPF), Weighted Frequency Fourier Linear Combiner (WFLC), Adaptive Frequency Estimator (AFE)]. BPF and AFE are the two most prevalent methods and they represent 38 and 15% of the tremor extraction algorithms, respectively. The cut-off frequencies used in BPF are (2–14 Hz) (Yi et al., 2019; Zahedi et al., 2020).

The two-stage methods account for 15% of the algorithms and found in systems, such as WOTAS (Rocon et al., 2007) and the Double Viscous Beam (DVB) (Loureiro et al., 2005). In the first stage, the Benedict-Bordner filter algorithm was used to estimate the voluntary movement. This algorithm implemented both estimation and filtering equations to filter out the tremorous movement and reduce the phase lags introduced by the filtering process. In the second stage, WFLC algorithm was applied to estimate tremor parameters including both the amplitude and time-varying frequency of the tremorous movement.

## 4. Conclusions

The results from our review reveal the efficacy of the biomechanical loading method using tremor-suppression orthoses in tremor suppression. Even though promising results have been demonstrated, the current systems remain bulky and heavy. These limitations significantly reduce the usability and wearability of the orthoses and create considerable barriers to the adoption from the users. Tremor-suppression orthoses require control algorithms that are able to detect tremorous movements with high accuracy and minimum time delay for intuitive interaction. However, the challenges in developing an intuitive control for tremor-suppression orthoses have not been fully addressed. Additionally, significant challenges for the translations of these systems into clinical or home use remain unsolved. Most of the devices in this review have only been evaluated in lab settings and are not commercially available. Therefore, we suggest future research to focus on improving the usability and wearability of the orthoses (e.g., compact, lightweight, aesthetic, and soft structure). Furthermore, the needs for intuitive and flexible user control interfaces (i.e., neural machine interface) should be addressed. Moreover, non-technical challenges (e.g., device cost, regulatory compliance, insurance reimbursement) need to be resolved to make the technology more accessible.

### 4.1. The Need for Lightweight, Soft Structure Tremor-Suppression Orthoses

Tremor-suppression orthoses based on passive and semi-active mechanisms attenuate tremorous motions by using shock absorbers, or adjusting the impedance of a damper attached to an upper limb. The efficacies of passive and semi-active orthoses have been reported in previous studies (Deuschl et al., 1998; Kotovsky and Rosen, 1998; Fromme et al., 2019). However, those systems not only suppress tremorous motions but also apply resistance and limit voluntary movements. Another drawback from the passive mechanism is that it cannot adapt to the changes of tremor dynamics. The inherent limitations from passive and semi-active mechanisms can be addressed by using active mechanisms. In particular, active orthoses are designed to provide motions opposing tremorous motions without suppressing voluntary movements. Tremor-suppression using active mechanisms is the most prevalent approach (Fromme et al., 2019). Our results from Figure 3B also show that active orthoses account for 47% of the devices, followed by semi-active (31%) and passive orthoses (21%). Two of the main barriers preventing the adoption of active orthoses are the size and weight of the devices. On average, an active tremor-suppression orthosis is about three times heavier than a passive one. The average weights of active and passive orthoses are 561 ± 467 and 191 ± 137 g, respectively (Figure 3B). Therefore, a wearer will be burdened with an additional weight of 18 ± 15% on the arm (data of human body segment masses from De Leva, 1996) while using an active orthosis. A bulky and heavy wearable device can lead to muscle fatigue (Rocon et al., 2014), discomfort, and is likely to be rejected by users. A study from Rocon et al. (2012) also reported that tremor-affected patients did not consider bulky exoskeletons as a solution to their problem due to the possibility of social exclusion.

Although biomechanical loading using active orthoses has demonstrated superior suppression efficacies, significant challenges remain (i.e., compact configuration, lightweight, aesthetic, and soft structure). Figure 3 shows that the majority of current active tremor-suppression orthoses are powered by DC motors (87.5%). Although DC motors are cost-effective and easy to control, they are usually heavy and rigid. The heaviest orthoses illustrated in Figure 4 are powered by DC motors (TSO: 1,600 g, WOTAS: 850 g). Minimizing the weight of a tremor-suppression orthosis that uses a traditional DC motor is challenging, especially for a system suppressing tremors at the elbow. A minimal system with only one DC motor and transmission developed by Ando et al. for tremor suppression at the elbow joint approximately weighs 330 g (Ando et al., 2010). Recent advances in soft actuators and artificial muscles could be promising solutions for developing a compact and lightweight tremor-suppression orthosis. For example, Pneumatic Artificial Muscles (PAM) have been gained extensive attention from researchers (Yang et al., 2019). PAMs can be lightweight, compact, and use flexible materials. They offer natural human-machine interaction and have been widely used in wearable assistive devices (Dzahir and Yamamoto, 2014). One of the drawbacks is that PAMs require an external air compressor which could be heavy and noisy. Other artificial muscles based on smart materials which are lightweight, soft, and quiet, could also be viable solutions (Lee et al., 2017; Mirvakili and Hunter, 2018). The artificial muscles could actuate based on thermally responsive methods [e.g., Shape Memory Alloys (SMAs)], electrically responsive methods (e.g, dielectric elastomers, electroactive polymers), and chemically responsive methods (e.g., hydrogels) (Bar-Cohen et al., 2017; Dong et al., 2018). Swallow and Siores (2009) introduced a conceptual design using Piezoelectric Fiber Composites (PFCs) to create a mechanically soft, lightweight glove that could be worn for tremor-suppression.

### 4.2. The Need for Intuitive Neural-Machine Interfaces

An intuitive and flexible user control interface plays a crucial role in developing a highly functional tremor-suppression orthosis. Unfortunately, this interface is still missing and remains one of the biggest challenges in the field. The results from Table 2 shows that 92% of the orthoses measured voluntary and/or tremorous motions using biomechanical sensors (e.g., IMU, force sensor). An intrinsic issue with the biomechanical sensors is that they can only measure the movements after the motor commands are executed, resulting in significant time-delays in the closed-loop control of a wearable orthosis. This delay was reported at ~100 ms after tremor onset (Yi et al., 2019; Zahedi et al., 2020). The time delay in assistive robots affects the intuitive control, decreases user comfort, and reduces the overall performance. For example, while the average efficacy of tremor-suppression orthoses was very high in bench testing at 95 ± 5% (Figure 5), the values were significantly lower when human subjects were involved (70 ± 12%). To maximize the potential benefits from tremor-suppression orthoses, an advanced neural interface and control framework capable of predicting motor commands, rather than responding to motor commands is required. In particular, sEMG can be used as a non-invasive interface to capture muscle activity in the upper limb. EMG-based neural interface and control has been demonstrated for the control of wearable exoskeletons (Kiguchi et al., 2007; Fleischer and Hommel, 2008; Artemiadis and Kyriakopoulos, 2010; Pau et al., 2012), and lower-limb prostheses (Au et al., 2008; Ha et al., 2010; Hargrove et al., 2015). Furthermore, sEMG was shown to be more informative than motion sensors, and can be used for real-time control of a prosthesis (Zhang and Huang, 2012). Recent studies have been exploring the potential of using EMG signals to extract tremor signals from voluntary commands (Ando et al., 2012; Matsumoto et al., 2012a, 2017). Prior studies have shown tremor signals modulated in the EMG as multiplicative noise instead of additive noise (Journee, 1983). This characteristic presents significant challenges for the real-time tremor extraction using EMG signals and the challenges have not been addressed yet (Matsumoto et al., 2012a,b, 2017).

An advanced neural interface using non-invasive Electroencephalography (EEG) could be a viable solution to optimize the intuitive control and efficacy in tremor-suppression orthoses. Even though none of the orthoses in this review used non-invasive EEG as a neural interface (Table 2), the potential of EEG in tremor estimation and control has been explored by Rocon et al. (2010). In their study, an EEG-based Brain-Computer Interface (BCI) system was designed to decipher tremor motor activities and provide high-level control signals to drive an FES system to suppress tremor motions. BCI system can provide direct control for assistive devices through non-muscular communication for individuals with motor-impairments. Recent studies have reported the feasibility of using non-invasive EEG-based BCI for the detection of motor intent (He et al., 2014; Kwak et al., 2015), and the prediction of continuous joint movements (Bradberry et al., 2010; Presacco et al., 2012; Robinson et al., 2015; Bhagat et al., 2016; Luu et al., 2016, 2017a,b; Nakagome et al., 2020). Future research directions in optimizing the control for tremor-suppression orthoses may involve a multimodal Neural Machine Interface (NMI) based on the fusion of EEG and EMG signals. The neural signals in a hybrid NMI will compliment each other and possibly provide faster and better estimates of voluntary and tremorous motions.

### 4.3. Commercial Challenges

Although research and development in tremor-suppression orthoses have shown promising results and a variety of devices have been developed and tested, the translation of these systems into clinical or home use is limited. To the best of our knowledge, none of the devices in this review are currently or have been commercially available. Both technical and non-technical challenges, such as technology adoptions, accessibility, cost, and risk assessment and mitigation need to be properly identified and addressed. Adoption of this technology could be enhanced by improving the wearability, usability, and functionality. Specifically, the wearable orthosis must be lightweight, mechanically flexible, easy to wear, visually appealing, and comfortable so that the users can wear them for ADLs with confidence. Additionally, clinical assessments are required to confirm the long-term benefits of biomechanical loading in tremor treatment using robotic orthoses.

To assure safety and effectiveness, a medical exoskeleton must comply with extensive governmental regulations relating to the design, development, manufacturing, software, labeling, and marketing of the products. In the USA, powered wearable orthoses could be regulated as medical devices under the Federal Food, Drug, and Cosmetic Act, or FD&C Act, as implemented and enforced by the FDA. Under the FDA regulations, a medical device is classified into Class I, Class II, or Class III, depending on the degree of risk associated with the device. Although the regulations may impose additional costs and reduce accessibility, they are critical to assure safety for the end-users. The cost of ownership of a powered exoskeleton is still incredibly expensive, including but not limited to the cost of the device, regulatory costs, and service and maintenance. The manufacturing of a medical device remains costly because of low-volume production, the limited numbers of contract manufacturers with expertise in the medical device industry, and inadequate numbers of certified third-party suppliers who can meet FDA's good manufacturing practice requirements for medical devices. The regulatory costs may substantially increase when a medical device is classified as either class II—with or without special controls—or class III. Additional expenses may include required clinical trials, more extensive mechanical and electrical testing, software testing, and other costs. The special controls of medical devices include (1) biocompatibility, (2) electromagnetic compatibility, electrical safety, thermal safety, mechanical safety, (3) software validation, (4) geometry and material composition, (5) various non-clinical performance testing, (6) clinical testing, (7) training program, and (8) labeling for the physician and user (Please see more details in Food and Drug Administration, 2014).

Another commercial challenge for a medical exoskeleton to successfully penetrate the market is insurance coverage. Medical device companies (e.g., ReWalk, Myomo) heavily rely on the sources of payment from private insurance plans, government programs, such as the Veterans Affairs (VA), Medicare and Medicaid, and other third-party payors. Unfortunately, obtaining and maintaining adequate insurance coverage or reimbursement for a medical exoskeleton is challenging. First, the market for medical exoskeletons remains relatively new and unproven. A uniform policy of coverage and reimbursement for powered medical exoskeletons is not yet available among third-party payors in the United States. Moreover, the cost control methods from third-party payors are sophisticated and potentially limit the amount that they may be willing to pay for clinical training using a medical exoskeleton, if they provide coverage at all.

## Data Availability Statement

The original contributions presented in the study are included in the article/supplementary material, further inquiries can be directed to the corresponding author/s.

## Author Contributions

TL contributed to the literature review, the collections of relevant articles, data analysis and interpretation, and manuscript draft. HN edited the manuscript and approved the final version. All authors contributed to the article and approved the submitted version.

## Conflict of Interest

The authors declare that the research was conducted in the absence of any commercial or financial relationships that could be construed as a potential conflict of interest.

## Abbreviations

ADL: activities of daily living
AFE: adaptive frequency estimator
BCI: brain-computer interface
BPF: band-pass filter
DBS: deep brain stimulation
DOFs: degrees of freedom
DRIFTS: dynamically responsive intervention for tremor suppression
DVB: double viscous beam
EEG: electroencephalography
EFE: elbow flexion/extension
FDA: Food and Drug Administration
FES: functional electrical stimulation
FPS: forearm pronation/supination
HPF: high-pass filter
IMU: inertial measurement unit
LPF: low-pass filter
MMS: multichannel mechatronic splitter
MR: magnetorheological
NMI: neural machine interface
PAM: pneumatic artificial muscles
PFCs: piezoelectric fiber composites
PRISMA: preferred reporting items for systematic reviews and meta-analysis
PSD: power spectral density
RMS: root mean square
sEMG: surface electromyography
SMAs: shape memory alloys
STFS: short-time Fourier transform
TAPO: task-adjustable passive orthosis
TDNN: time delay neural network
TSO: tremor suppression orthosis
VA: veterans affairs
WFE: wrist flexion/extension
WFLC: weighted frequency Fourier linear combiner
WOTAS: wearable orthosis for tremor assessment and suppression
WRU: wrist radial/ulnar deviation
WTSE: wrist tremor suppression exoskeleton
WTSG: wearable tremor suppression glove.
